# Comparing the Effect of Isoinertial Flywheel Training and Traditional Resistance Training on Maximal Strength and Muscle Power in Healthy People: A Systematic Review and Meta-Analysis

**DOI:** 10.3390/life14070908

**Published:** 2024-07-21

**Authors:** Zhongzhong Hu, Yuhang Liu, Keke Huang, Hao Huang, Feng Li, Xiaoyi Yuan

**Affiliations:** 1 School of Sports Science, Wenzhou Medical University, Wenzhou 325035, China; bsu2014hzz@163.com (Z.H.); huangkekerunning@163.com (K.H.); huanghaomedical@163.com (H.H.); lifengball@163.com (F.L.); 2 China Athletics College, Beijing Sport University, Beijing 100084, China; 15031958573@163.com

**Keywords:** eccentric overload training, isoinertial flywheel training, maximal strength, muscle power

## Abstract

Background: This systematic review and meta-analysis aimed to analyze whether isoinertial flywheel training (FWT) is superior to traditional resistance training (TRT) in enhancing maximal strength and muscle power in healthy individuals. Methods: Electronic searches were conducted in the Web of Science, PubMed, Cochrane Library, SPORTDiscus, and Scopus databases up to 21 April 2024. Outcomes were analyzed as continuous variables using either a random or fixed effects model to calculate the standardized mean difference (SMD) and 95% confidence intervals (CI). Results: A total of sixteen articles, involving 341 subjects, met the inclusion criteria and were included in the statistical analyses. The pooled results indicate no statistically significant differences between FWT and TRT in developing maximal strength in healthy individuals (SMD = 0.24, 95% CI [−0.26, 0.74], *p* = 0.35). Additionally, the pooled outcomes showed a small-sized effect in muscle power with FWT (SMD = 0.47, 95% CI [0.10, 0.84]), which was significantly higher than that with TRT (*p* = 0.01) in healthy individuals. Subgroup analysis revealed that when the total number of FWT sessions is between 12 and 18 (1–3 times per week), it significantly improves muscle power (SMD = 0.61, 95% CI [0.12, 1.09]). Significant effects favoring FWT for muscle power were observed in both well-trained (SMD = 0.58, 95% CI [0.04, 1.13]) and untrained individuals (SMD = 1.40, 95% CI [0.23, 2.57]). In terms of exercise, performing flywheel training with squat and lunge exercises significantly enhances muscle power (SMD = 0.43; 95% CI: 0.02–0.84, and *p* = 0.04). Interestingly, FWT was superior to weight stack resistance training (SMD = 0.61, 95% CI [0.21, 1.00]) in enhancing muscle power, while no significant differences were found compared to barbell free weights training (SMD = 0.36, 95% CI [−0.22, 0.94]). Conclusions: This meta-analysis confirms the superiority of FWT compared to TRT in promoting muscle power in both healthy untrained and well-trained individuals. Squats and lunges for FWT are more suitable for improving lower limb explosive power. It is recommended that coaches and trainers implement FWT for six weeks, 2–3 times per week, with at least a 48 h interval between each session. Although FWT is not superior to free weights training, it is advisable to include FWT in sport periodization to diversify the training stimuli for healthy individuals.

## 1. Introduction

The maintenance and improvement of muscle strength and power are critical goals of physical training interventions across various populations, with resistance training emerging as the most prevalent method for achieving these outcomes [1,2]. Over the years, numerous methods to enhance strength were suggested, including the use of free weights, weight stacks, resistance bands, flywheels, and pneumatic resistance machines [3]. Traditional resistance training (TRT) involving free weights and weight stack machines, which rely on gravity-dependent loads, were demonstrated to elicit desirable structural and neural adaptations in healthy individuals [4]. In conventional setups, the external load provided by resistance equipment remains static throughout the entire range of motion. Eccentric muscle contractions, however, allow for higher force production than concentric contractions [5,6], the relative load during the eccentric phase in constant resistance training is inadvertently lower than in the concentric phase. This discrepancy leads to suboptimal loading, resulting in reduced motor unit recruitment and firing rates during the eccentric phase [7], potentially causing diminished sarcoplasmic calcium release and, consequently, a lesser stimulus for myocellular adaptation [8]. As a result, this traditional approach may not provide an optimal stimulus for the critical eccentric phase.

Eccentric overload training (EOT) can reduce skeletal muscle resistance in the weakest areas of motion, provide greater resistance in stronger areas, and align more closely with human strength curves to enable muscles to function over a broader range [9,10,11]. While EOT can be implemented using gravity-dependent (GD) devices, these often require third-party assistance, posing a limitation in various settings. Historically, practitioners utilized weight releasers in traditional training methods to achieve supramaximal eccentric loads, although this approach still presents certain constraints. This form of resistance training effectively reduces the mechanical disadvantage of the sticking point commonly encountered in free weight training [12,13]. Recognizing these limitations, several innovative methods emerged, including the adoption of non-gravity-dependent technology. Specifically, isoinertial flywheel devices, which harness the inertia of a rotating wheel and the subsequent stored kinetic energy, offer a higher eccentric load compared to traditional weight training methods [14,15]. Although no differences were found in muscle fatigue levels, flywheel training (FWT) induced greater physiological stress than barbell squat training. This was observed through a greater decrease in muscle oxygen saturation and a longer reoxygenation period [16].

Scientific literature acknowledges that FWT induces several morphological and neural-adaptive changes in the human body. These include increases in peak power output, muscle cross-sectional area, musculotendinous stiffness, as well as improvements in motor unit recruitment, rate coding (firing frequency), synchronous motor unit activity, and neuromuscular inhibition [17,18]. In contrast to TRT, this form of accentuated eccentric training induces a prolonged eccentric strain, which may lead to superior adaptations. Prolonged eccentric training appears to increase eccentric kinetic energy and enhance performance more effectively than traditional methodologies [19]. Inertial technology emerged as an alternative that enables accentuating eccentric overload in more specific sports actions, such as changing direction. This is essential for player optimization, reducing the risk of injuries, and aiding in injury rehabilitation [20]. While studies confirm that FWT can yield more acute [21,22,23] and long-term [24,25,26] training effects on strength performance than traditional constant resistance training, some authors report no significant difference between the two methods [27,28]. A limitation of existing studies lies in the use of notably different protocols and execution methodologies. For instance, variations in training methods, targeted muscle groups, sets and repetitions performed, measurement tools, eccentric load applied, participants’ age, and training experience differ significantly among studies. Nonetheless, our analysis builds on previous meta-analyses by incorporating a larger number of studies, more recently published data, and comparative analyses on the effects of FWT on different training levels of participants, and inertial flywheel training compared to free weights or weight stack training. The primary aim of this meta-analysis was to compare the effects of FWT versus TRT on muscle power and maximal strength by examining and compiling relevant studies.

## 2. Methods

This meta-analysis was conducted in accordance with the Preferred Reporting Items for Systematic Reviews and Meta-analyses (PRISMA) statement guidelines [29]. Prior to the search, a review protocol was registered at PROSPERO (ID = CRD42023491903).

### 2.1. Search Strategy

The electronic databases PubMed, SPORTDiscus, Web of Science, Cochrane Library, and Scopus were searched for randomized controlled trials on flywheel training from their inception to 21 April 2024. Search terms included: ‘eccentric overload training’, ‘flywheel training’, ‘flywheel resistance training’, ‘flywheel exercise’, ‘isoinertial training/inertial training’, ‘isoinertial exercise/inertial exercise’, ‘strength’, and ‘power’. Boolean operators ‘AND’ and ‘OR’ were used to combine key search terms. Using the WOS database for example, (AB = (flywheel training OR flywheel resistance training OR flywheel exercise OR isoinertial training OR inertial training OR isoinertial exercise OR inertial exercise)) AND (AB = (strength OR power)). When applicable, filters were used during the initial literature search to identify relevant articles. A hand-search of the reference lists of relevant articles was also conducted for other potentially relevant references.

### 2.2. Inclusion/Exclusion Criteria

To rate studies for eligibility, a participants, intervention, comparators, study outcomes, and study design (PICOS) approach was used [30]. An article was eligible for inclusion if it met all of the following criteria: (1) the original article was a randomized controlled trial (RCT); (2) participants were healthy, with no imposed limitations concerning gender, training status, sport specialty, or body composition; (3) the manuscript included an FWT intervention and a control or alternative intervention group aimed at evaluating training adaptations in strength and/or power; (4) the article stipulated that participants completed an FWT protocol lasting at least four weeks; and (5) the study provided data on at least one of the following outcome measures: strength (e.g., 1 RM, maximal voluntary contraction, and peak torque) and power (e.g., jump height, rate of force development, and peak power).

An article was excluded if it met any of the following criteria: (1) it was a non-randomized controlled trial; (2) it failed to meet the minimum requirements for the training protocol (e.g., duration or frequency); (3) the document was a literature review, abstract, editorial commentary, or letter to the editor; (4) it was not written in English; (5) means and standard deviations were not reported, and the authors did not respond to our inquiries; and (6) the study involved participants with any pathology or those receiving treatment for musculoskeletal injuries in the trained limb.

Titles and abstracts identified in the search were downloaded into EndNote 20, after which cross-references and duplicates were deleted. All publications potentially relevant for inclusion were independently assessed by two reviewers, with full texts obtained if necessary. Any discrepancies that arose were resolved during a consensus meeting, with the provision that a third reviewer was available if needed.

### 2.3. Study Coding and Data Extraction

Two reviewers independently extracted data using a specially designed standardized form, focusing on general study information, participant demographics, intervention characteristics, and outcome measures. If the necessary data were not explicitly available in tables or the text’s results section, the first author of the systematic review proactively contacted the original authors to request the missing data. When the authors did not have access to their data, essential details, such as means and standard deviations for outcome measures, were meticulously extracted from figures and graphs using Web Plot Digitizer V4.7 software. To ensure accuracy, another reviewer then rigorously verified the validity of the data extraction.

Each article was read and coded by two investigators focusing on several variables: (a) descriptive information, such as participants’ details (age, body mass, and height), physical activity status (trained or untrained), sex, and the total number of participants; (b) specifics of the program exercises, including the type of exercise (knee extension, squat, half-squat, leg press, deadlift, and bench press); (c) program variables, detailing the frequency of weekly sessions, duration of the training period, total number of sessions, number of sets per session, number of repetitions per set, and training intensity; and (d) outcome measurements, capturing measures of maximal muscle strength and/or muscle power. The investigators’ mean agreement was quantified using an intraclass correlation coefficient (ICC), with the coding agreement assessed by comparing the number of variables on which they aligned versus the total coded. A mean agreement of 0.90 is upheld as an appropriate level of reliability for such coding procedures [31]. Any discrepancies in coding between investigators were meticulously scrutinized and resolved before proceeding with the analysis.

### 2.4. Quality Assessment

Two investigators conducted independent quality assessments of the included studies, with any disagreements resolved through a consensus meeting mediated by a third party. We used the 2019 Cochrane Risk of Bias 2 tool (RoB2) to assess the risk of bias across five domains: randomization process, deviations from intended interventions, missing outcome data, measurement of the outcome, and selection of the reported result [32]. Each of the 16 studies included in the quantitative analysis was longitudinal. Two independent authors assessed the quality using checklists. If there was a disagreement regarding the risk of bias assessment findings, a third reviewer was consulted to evaluate the data and make the final decision.

### 2.5. Statistics and Data Analysis

Stata 17.0 and Reviewer Manager 5.4 software were instrumental for various tasks, including data merging, subgroup analysis, forest plot generation, heterogeneity analysis, meta-regression, and assessing publication bias. For the primary outcome focusing on muscle strength and power, we calculated intervention effects using standardized mean differences (SMD) with 95% confidence intervals (CI), which is appropriate due to the continuous nature of the data. Effect sizes were stratified as small (0.2), medium (0.5), or large (0.8 or greater) [33]. We employed the I^2^ test to examine the heterogeneity of each trial, with benchmarks set at 25%, 50%, and 75% for low, medium, and high statistical heterogeneity, respectively. The chi-squared and I^2^ statistics were pivotal in describing the level of heterogeneity or homogeneity among the comparisons, with a *p*-value threshold of less than 0.05 indicating significant heterogeneity [34]. In cases where the heterogeneity test showed no significant differences, a fixed-effects model was adopted for the meta-analysis. Conversely, a random effects model was applied in the presence of high heterogeneity. We conducted a detailed subgroup analysis to identify and analyze potential sources of heterogeneity.

## 3. Results

### 3.1. Study Selection

**Figure 1:**
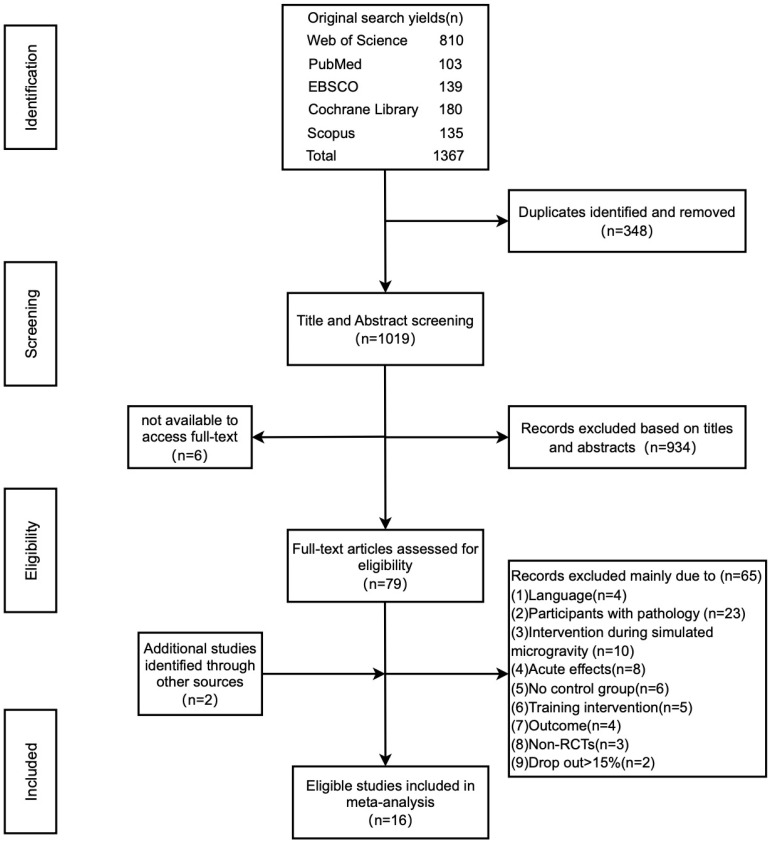
Flow chart illustrating the selection process for all included and excluded studies.

A search of electronic databases, along with scanning the reference lists, yielded 1367 relevant studies. After removing duplicates, 1019 titles and abstracts were screened. From these, 934 records were excluded based on their titles and abstracts, and 6 records were excluded due to the unavailability of full text. This led to the selection of 79 studies, which were then carefully screened for eligibility. During this process, two additional records were identified through meticulous examination of reference lists and citations of pertinent articles. A total of 65 studies were excluded for the following reasons: (1) 3 studies were published in German and 1 study was published in Korean; (2) the participants in 23 studies had diseases or were injured; (3) 10 studies performed interventions during simulated microgravity; (4) 8 studies focused on acute effects; (5) 6 studies did not have a control group; (6) the control group in 5 studies did not use free weights or weight stack training as the intervention; (7) the outcome measures in 4 studies did not include maximal strength and power; (8) 3 studies were not RCT designs; and (9) the dropout rate was greater than 15% in 2 studies. Ultimately, 16 studies met the inclusion criteria and were included in the meta-analysis. The detailed flowchart in Figure 1 illustrates the systematic selection process of the studies.

### 3.2. Descriptive Characteristics of the Studies

The main characteristics of the studies included in the review, encompassing participants, interventions, and results, are depicted in Table 1. Following an adjustment for dropouts, the total number of participants in the 16 studies was 341. Of these 341 participants, 171 undertook FWT, while the remaining 170 engaged in free weights training or weight stack training. The estimated average ages of the experimental and control groups were 32.18 ± 16.68 and 34.01 ± 18.51, respectively. Notably, the distribution of genders across the studies was imbalanced, with only three studies incorporating female participants, culminating in a demographic of just 32 women and 309 men. The participants in three studies were novices in resistance training, lacking or having scant experience in this discipline. Conversely, thirteen studies involved subjects with prior strength training experience: five studies included seasoned athletes engaged in professional or semi-professional leagues, and eight involved individuals who trained recreationally (two with college students active in sports, three involving strength-trained individuals, and three focusing on junior athletes).

In the experimental groups, all studies utilized inertial flywheel devices for eccentric overload (EO). Regarding the control groups, eight studies implemented free weights training, while eight opted for resistance training with a weight stack machine. The duration of training interventions varied from 4 to 12 weeks, with participants undertaking an average of 2.3 ± 0.7 sessions weekly, culminating in 17.5 ± 11.5 sessions per study. Among the studies, there was variation in the total number of sets (3–7) and repetitions (4–12) per session. Inertia was a key variable, with eleven studies employing a range from 0.0291 to 0.145 kg·m^2^; however, five studies did not disclose the inertia used. The most commonly used load intensity for the control group was 80% 1 RM or 7 RM.

### 3.3. Quality of the Selected Studies

**Figure 2:**
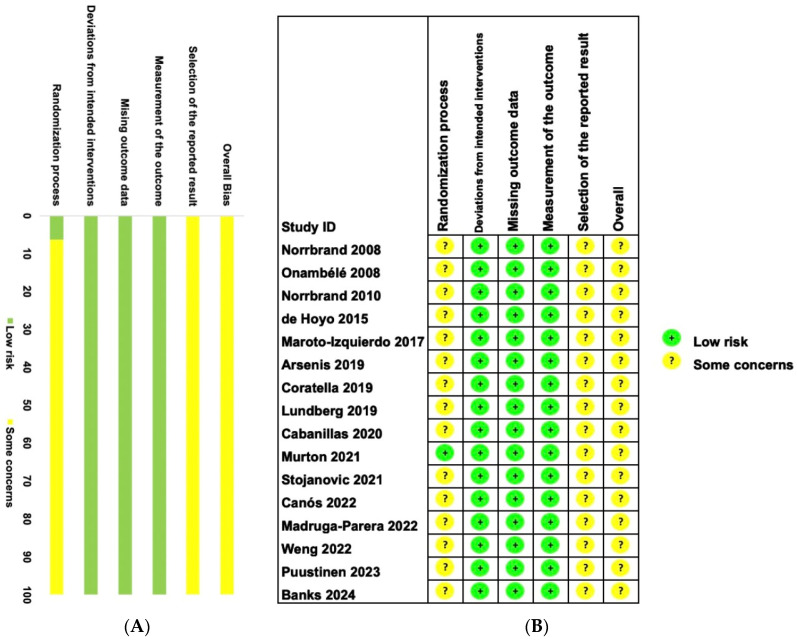
Summary of the risk of bias of studies included in this meta-analysis [35,36,37,38,39,40,41,42,43,44,45,46,47,48,49,50]. (**A**) Summary of 16 studies in six different domains of bias. (**B**) Details of 16 studies in six different domains of bias.

Assessing the risk of bias was conducted using a revised version of the Cochrane Risk of Bias (RoB 2) tool, evaluating individual studies across six different domains of bias (Figure 2A). Only one study [45] reported randomization sequence generation. All sixteen studies included in the meta-analysis were rated as having “some concerns” for risk of bias (Figure 2B), likely because it is impossible to blind subjects to experimental and control groups in studies of this nature. Nevertheless, it is important to acknowledge that blinding participants is a notably challenging criterion to meet in this context. Adding to the credibility of these assessments, the studies demonstrated significantly high inter-rater reliability (ICC = 0.95).

### 3.4. Publication Bias and Sensitivity

**Figure 3:**
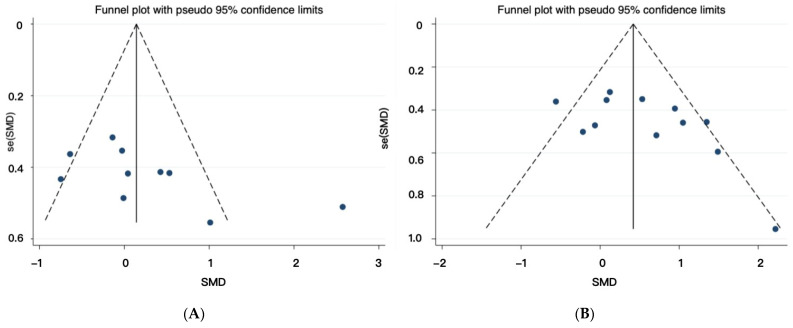
Funnel plot illustrating the symmetrical distribution of the effects across the included studies. (**A**) Funnel plot for studies about maximal muscle strength. (**B**) Funnel plot for studies about muscle power.

A funnel plot visually represents each individual study’s effect by considering the study size in relation to the difference observed between pre- and post-tests. A symmetrical funnel plot, centered around the mean effect of the collective studies, indicates that the identification and selection processes are likely free from bias [51]. The corresponding funnel plots are illustrated in Figure 3A,B. The visual inspection of the funnel plot indicates a symmetrical distribution pattern of the effects, illustrating the absence of publication bias. This is corroborated by Egger’s regression outcome, which also indicates that the distribution pattern of the effect in the funnel plot is symmetrical (maximal muscle strength: t = 2.06, *p* = 0.073; muscle power: t = 2.13, *p* = 0.059).

**Figure 4:**
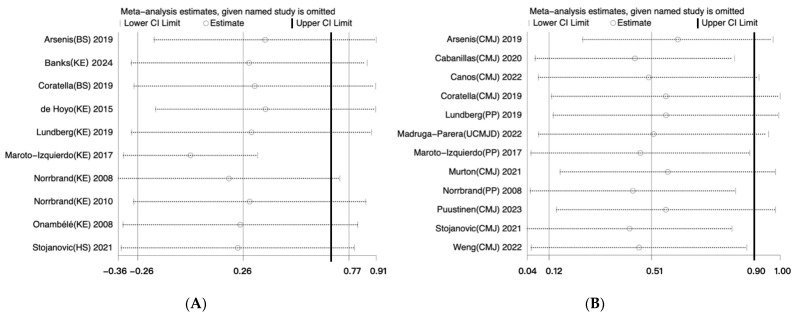
Sensitivity analysis for this meta-analysis [35,36,37,38,39,40,41,42,43,44,45,46,47,48,49,50]. (**A**) Sensitivity analysis for studies about maximal muscle strength. (**B**) Sensitivity analysis for studies about muscle power.

In a separate sensitivity analysis, we assessed the contribution of each study to the overall improvement in maximal muscle strength (Figure 4A) and muscle power (Figure 4B) detected in this meta-analysis. This was achieved by successively omitting the results of each study from the comparisons made with the data from the remaining studies. In each scenario where the results of one study were omitted, no significant differences were detected, indicating the robust contribution of all the studies to the observed gains in maximal muscle strength and muscle power.

### 3.5. Main Analysis

#### 3.5.1. Meta-Analysis Results on Muscle Power

**Figure 5:**
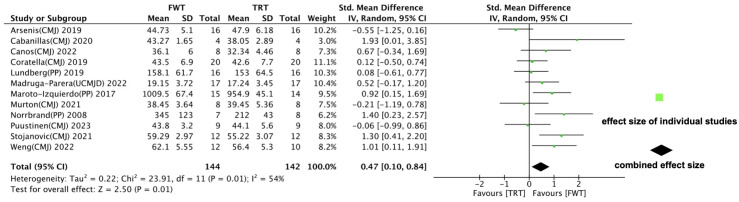
Forest plot with meta-analysis of standardized mean difference showing comparison of flywheel training (FWT) versus traditional resistance training (TRT) on muscle power [36,39,41,42,43,44,45,46,47,48,49].

A total of twelve reports were included in the meta-analysis. The data presented in Figure 5 reveal significant differences between FWT and TRT in improving the muscle power of healthy subjects (ES = 0.47; 95% CI: 0.10–0.84, *p* = 0.01). However, our analysis detected moderate statistical heterogeneity (I^2^ = 54%).

#### 3.5.2. Meta-Analysis Results on Maximal Strength

**Figure 6:**
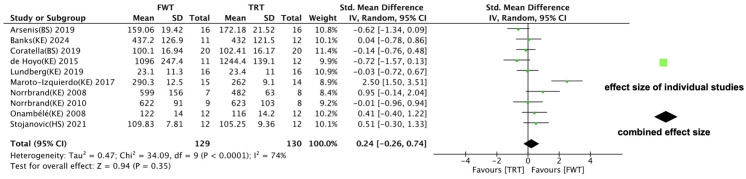
Forest plot with meta-analysis of standardized mean difference showing comparison of flywheel training (FWT) versus traditional resistance training (TRT) on maximal strength [35,36,37,38,39,40,41,42,44,50].

Ten of the included reports examined the effects of FWT versus TRT on maximal muscle strength, with measures encompassing 1 RM, peak torque, and maximal voluntary contraction. The analysis (Figure 6) revealed no significant differences in outcomes between the two training modalities (ES = 0.24; 95% CI: −0.26–0.74, *p* = 0.35), although a high degree of heterogeneity was observed (I^2^ = 74%).

### 3.6. Subgroup Analysis

#### 3.6.1. Subject-Related Moderating Variables

**Figure 7:**
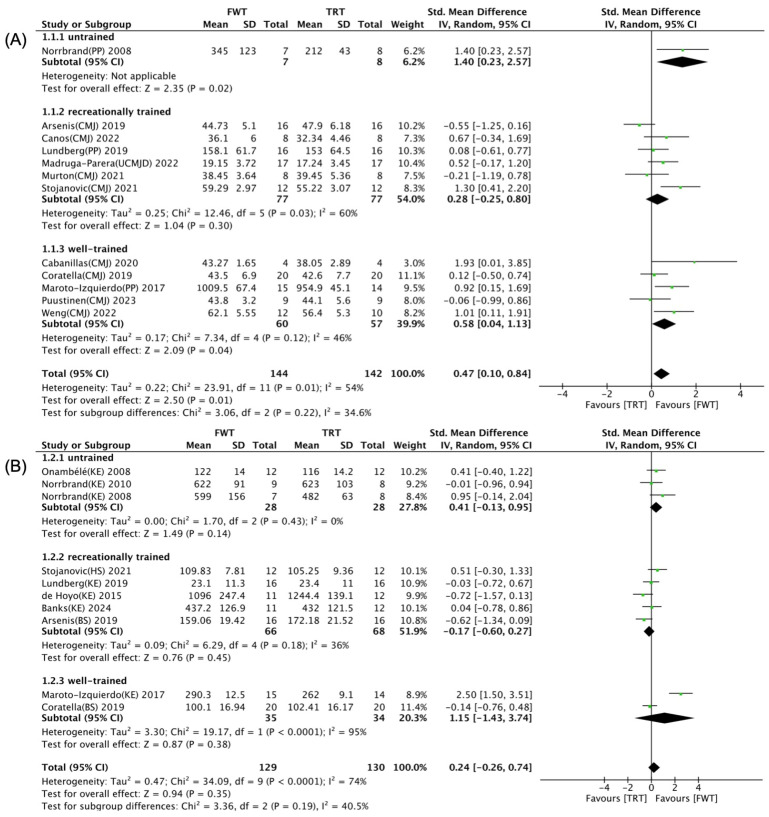
Forest plot with subgroup analysis of strength training experience [35,36,37,38,39,40,41,42,43,44,45,46,47,48,49,50]. (**A**) Effect of strength training experience on muscle power. (**B**) Effect of strength training experience on maximal muscle strength. 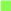 effect size of individual studies. 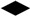 combined effect size.

The impact of strength training experience on the differential effects of FWT versus TRT on maximal muscle strength and muscle power is illustrated in Figure 7. A univariate subgroup analysis indicated that strength training experience did not significantly influence the FWT/TRT effects on maximal muscle strength (*p* = 0.35, Figure 7B), while it did play a crucial role in modulating the effects on muscle power (*p* = 0.01, Figure 7A). There were significant and moderate-sized effects in favor of FWT over TRT for muscle power among well-trained individuals (SMD = 0.58, *p* = 0.04), alongside large-sized effects observed for muscle power in untrained individuals (SMD = 1.40, *p* = 0.02). However, for maximal muscle strength, there were no notable differences between FWT and TRT in both untrained and trained participants (*p* > 0.05).

#### 3.6.2. Training-Related Programming Parameters

**Figure 8:**
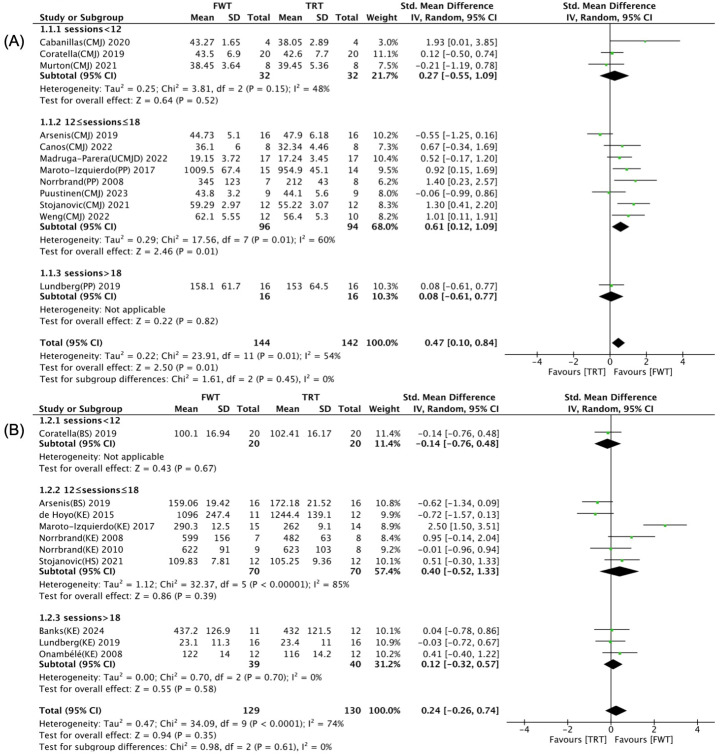
Forest plot with subgroup analysis of total number of training sessions [35,36,37,38,39,40,41,42,43,44,45,46,47,48,49,50]. (**A**) Effect of total number of training sessions on muscle power. (**B**) Effect of total number of training sessions on maximal muscle strength. 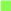 effect size of individual studies. 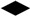 combined effect size.

**Figure 9:**
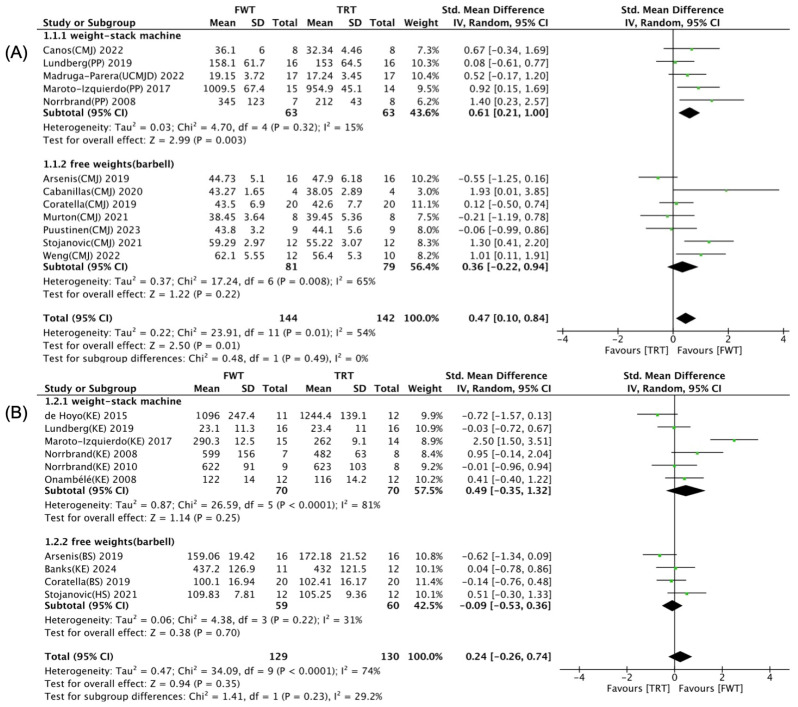
Forest plot with subgroup analysis of control group’s intervention [35,36,37,38,39,40,41,42,43,44,45,46,47,48,49,50]. (**A**) Effect of control group’s intervention on muscle power. (**B**) Effect of control group’s intervention on maximal muscle strength. 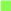 effect size of individual studies. 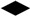 combined effect size.

**Figure 10:**
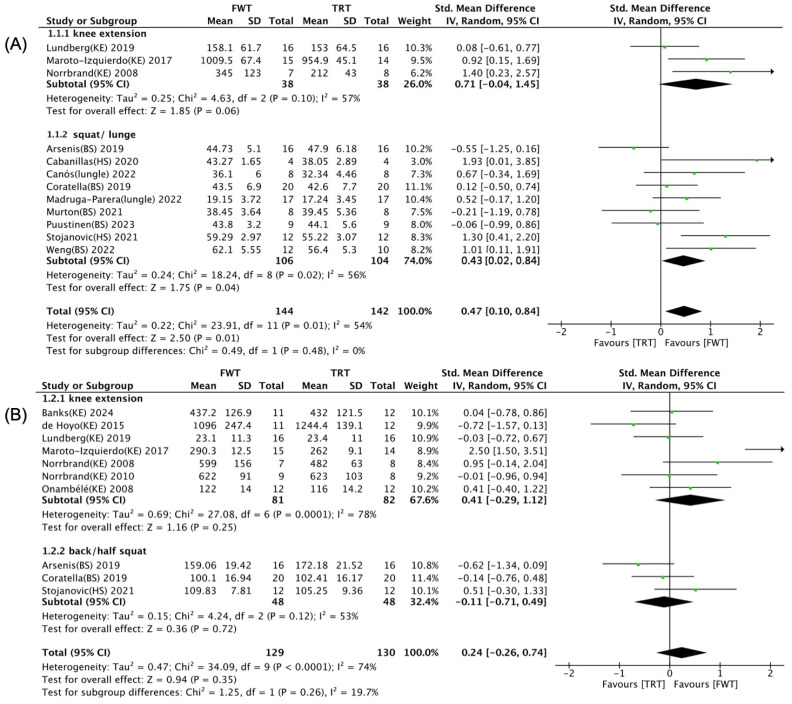
Forest plot with subgroup analysis of selected exercise [35,36,37,38,39,40,41,42,43,44,45,46,47,48,49,50]. (**A**) Effect of selected exercise on muscle power. (**B**) Effect of selected exercise on maximal muscle strength. 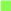 effect size of individual studies. 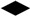 combined effect size.

The effects of training-related programming parameters for FWT/TRT on maximal muscle strength and muscle power are illustrated in Figure 8, Figure 9 and Figure 10. Univariate subgroup analyses highlighted that the total number of training sessions, the type of control group intervention, and the selected exercise significantly influenced the impact of FWT versus TRT on muscle power, with notable distinctions based on these variables (*p* < 0.05). As shown in Figure 8A, a significant and moderate enhancement of muscle power was noted with FWT for those undertaking 12–18 training sessions (SMD = 0.61; 95% CI: 0.12–1.09, *p* = 0.01), contrasting with a lack of such improvement for schedules with fewer than 12 or more than 18 sessions (*p* > 0.05). Furthermore, muscle power significantly increased (Figure 9A) when comparing FWT with weight stack training (SMD = 0.61; 95% CI: 0.21–1.00, *p* = 0.003), unlike its free weight training counterpart (*p* = 0.22). According to Figure 10A, performing flywheel training with squat and lunge exercises significantly enhances muscle power (SMD = 0.43; 95% CI: 0.02–0.84, *p* = 0.04). However, when using knee extension exercises, there is no significant difference between FWT and TRT (*p* = 0.06).

Regarding the total number of training sessions (Figure 8B), there were no significant differences between the two training modalities in maximal muscle strength, regardless of whether participants undertook 12–18 sessions or fewer than 12 or more than 18 sessions (*p* > 0.05). In terms of the control group’s intervention (Figure 9B), no significant differences were detected in maximal muscle strength when performing weight stack or free weights training as the control intervention (*p* > 0.05). Regardless of whether single-joint or multi-joint training exercises are selected, there is no statistically significant difference between FWT and TRT in enhancing maximum muscle strength (*p* > 0.05). However, as shown in Figure 10B, selecting knee extension exercises during FWT is more conducive to maximum strength gains (SMD = 0.41; 95% CI: −0.29–1.12), while choosing squat exercises during TRT yields better results for maximum strength (SMD = −0.11; 95% CI: −0.71–0.49).

## 4. Discussion

### 4.1. Main Analysis

This study systematically synthesized and quantified existing evidence, comparing the effects of FWT versus TRT on muscle power and maximal strength. Sixteen studies meeting the inclusion criteria were analyzed, with twelve studies assessing muscle power and ten studies focusing on maximal muscle strength. This meta-analysis included only studies involving healthy individuals, both untrained and trained, further subdivided into well-trained and recreationally trained categories. The results indicate a small but significant increase in muscle power with FWT (ES = 0.47; 95% CI: 0.10–0.84, *p* = 0.01) compared to TRT. From a molecular perspective, FWT appears to increase mRNA levels of genes expressed predominantly in fast glycolytic fibers, potentially inducing a faster muscle phenotype. This adaptation likely optimizes muscle for explosive, high-speed actions [52]. Furthermore, our analysis revealed no significant difference between FWT and TRT concerning maximal strength (*p* > 0.05). Despite extensive research elucidating the benefits associated with FWT, discrepancies persist regarding its efficacy on maximal strength. A previous meta-analysis encompassing seven studies on FWT concluded that it does not offer clear advantages over TRT in enhancing maximal strength [28]. Conversely, De Keijzer provides a contrasting perspective, affirming the effectiveness of FWT in boosting maximal strength across both healthy and athletic cohorts. De Keijzer’s synthesis of 11 pertinent reviews posits FWT as a viable alternative to conventional resistance training, citing improvements in muscular strength, power, and jump performance among diverse population groups [53]. Nevertheless, variables across studies, such as exercise selection, instructional nuances (e.g., delayed eccentric action), training background, frequency, and duration, necessitate careful consideration in interpreting these results.

### 4.2. Subgroup Analysis

A limitation of existing studies lies in the use of notably different protocols and execution methodologies. For instance, variations in training methods, targeted muscle groups, sets and repetitions performed, and training experience differ significantly among selected studies. However, our study overcame this limitation by conducting subgroup analysis. By categorizing and separately analyzing different variables, such as total number of training sessions, participants’ strength training experience, training methods, and selected exercise, we were able to more accurately assess the impact of these factors on training outcomes.

#### 4.2.1. Total Number of Training Sessions

FWT is a unique training mode gaining popularity in the research community. However, the optimal duration of an FWT program for athletes to develop sufficient neural and muscular adaptations, thereby enhancing maximal strength and power, remains elusive. Our meta-analysis, encompassing multiple studies with varied protocols, indicates that 12–18 sessions of FWT result in significantly greater power gains compared to TRT. These findings not only corroborate those reported by Sanchez et al. [54], but also demonstrate increased statistical power (from 103 to 208 participants) and a heightened magnitude of strength performance change, from 0.21 (small) to 0.61 (moderate). FWT was documented to cause subcellular damage to the contractile and structural components of skeletal muscle, inducing local and systemic inflammatory responses [55,56,57]. Consequently, an overly brief intervention period might involve a recovery process, potentially leading to deteriorated sport performance due to fatigue [58]. Moreover, participants typically require two or three familiarization sessions to acclimate to the training apparatus and techniques. FWT programs encompassing 12–18 sessions yielded favorable outcomes [37,39,46] as they afford sufficient recovery and over-compensation periods, which is advantageous for muscle performance. Conversely, excessively prolonged interventions may trigger physiological and neural adaptations, or even training burnout, hindering further improvement or even causing regression in sports capabilities [59]. Notably, eccentrically induced muscle damage can alter resting metabolic rates for up to 48 h post-exercise [60,61,62]. Buonsenso et al. also suggested that 2–3 times per week of FWT is an optimal frequency to improve jump performance [18]. Thus, we recommend coaches and trainers allocate a 6-week training block, comprising 2–3 weekly sessions, each followed by 48 h of recovery, to foster optimal muscle performance through FWT. To avoid training adaptations resulting from long-term FWT, it is recommended to increase the moment of inertia or shorten rest intervals to enhance training intensity when the total number of sessions exceeds 18.

#### 4.2.2. Strength Training Experience

When subjects were stratified according to training experience, untrained and well-trained individuals achieved significantly greater power gains with FWT than with TRT. It should be highlighted that subjects labeled as “well-trained” in our study are typically considered “elite athletes,” participating in professional or semi-professional leagues. Suarez-Arrones et al. found similar results, applying FWT to elite soccer players throughout an entire competitive season, which significantly improved half squat power output [63]. Although only one study on the untrained population met the criteria for inclusion in the subgroup analysis, Sáez-Michea demonstrated that strength training with the isoinertial method effectively improves CMJ jump ability, running velocity, and dynamic postural balance in healthy untrained adults [64]. Due to the limited number of studies, further high-quality investigations are necessary to confirm current findings. In addition, the power increment observed for recreationally trained subjects undertaking an FWT program versus a traditional program did not vary significantly (ES = 0.28; 95% CI: −0.25–0.80, *p* = 0.30). In our analysis, “recreationally trained” refers to participants other than well-trained athletes with more than one year of strength training experience. As consistently reported in the literature, there is no significant increase in eccentric hamstring strength following a six-week flywheel leg curl protocol [65]. These results are inconsistent with those of Allen, who noted that diverse FWT interventions can effectively improve strength, power, and jump performance in male soccer players of varying levels [66]. Differences in maximum neural activation and recovery ability between training sessions of athletes of different technical levels may explain the differences in power outcomes. Another explanation could be that well-trained athletes, having more familiarity with flywheel tempo, engage more actively in both concentric and eccentric actions, thereby achieving greater power gains. Indeed, the enhanced eccentric overload generated during isoinertial FWT is generally more pronounced in individuals with prior strength experience, underscoring the necessity of proper technique to optimize this training method’s benefits [67]. Thus, it is possible to conclude that well-trained athletes may be able to reap greater benefits from FWT than recreationally trained individuals.

In our analysis, we also examined the effects of TRT versus FWT on maximal muscle strength among subjects with different training backgrounds. However, no significant differences were detected between TRT and FWT in either trained or untrained populations. A previous review confirmed that FWT is a valid strategy to improve strength; however, differences with traditional training programs were not clearly established, making it impossible to state that FWT is superior to TRT methodologies [68]. Flywheel eccentric overload training protocols did not improve lower-body one-repetition maximum (1 RM) more effectively than traditional training methods, but the evidence is insufficient due to decreased compliance with the intervention, which was connected to the effects of delayed onset muscle soreness [69]. Interestingly, Sagelv et al. reported improvement in maximal squat strength in amateur soccer players, with more significant gains after a traditional squat protocol than with a FWT program [70]. However, the dropout rate of 18.75% in this study may reduce the credibility of the results. Such discrepancies in reported outcomes may stem from variations in exercise selection and loading parameters used in each study. Our meta-analysis revealed that FWT is a favorable strategy for improving power in both elite athletes and untrained individuals, presenting clear implications for coaches and sports science specialists. However, it is pertinent to acknowledge that this meta-analysis encompassed a limited number of studies. Therefore, conducting further robust research is essential for a more comprehensive understanding of performance adaptations following FWT interventions. 

#### 4.2.3. Control Group’s Intervention

In our meta-analysis, the control group interventions included barbell free weights training and weight stack training. Based on the available data, inertial flywheel resistance training was superior to weight stack resistance training in enhancing muscle power, while no significant differences were detected when comparing it to barbell free weights training. Our findings confirm those of the study by Alkner, which concluded that quadriceps muscle activation was superior in flywheel exercise compared to weight stack knee extension exercise [71]. This outcome aligns with the findings of Nunez Sanchez, who noted that FWT provided additional benefits to muscle strength compared with knee extension machines [54]. In contrast, Raya-Gonzalez identified no differences between inertial flywheel and barbell free weights training in muscle strength [68]. This discrepancy may be due to isolated single-joint movements not fully exploiting the stretch-shortening cycle, whereas multi-joint exercises promote the recruitment of a greater number of motor units. Both free weight training and FWT share the benefit of activating more muscle groups, which is not possible with weight stack training. Allen suggested that enhanced utilization of elastic potential energy during the stretch-shortening cycle (SSC) and increased muscle–tendon unit stiffness from FWT could significantly boost jump performance [66]. Similarly, strength augmentations potentially arise from neural adaptations, such as heightened neural drive, modified motor unit firing rates, and improved motor unit synchronization, all of which might be amplified by multi-joint movements [62,72]. However, some meta-analyses generated contradictory results, showing more significant improvements in maximal strength and power output with inertial flywheel resistance training than with gravity-dependent resistance training [52,66,73,74]. These differences could be due to heterogeneity between participants or differences in training volume, rest intervals, and the inertia of the flywheels used in each study. A range of inertial intensities (0.025–0.11 kg·m^2^) are generally recommended to induce chronic adaptations and enhance athletic performance [41,75]. It is found that higher inertial intensities may be preferable for developing force, while lower inertial intensities could be used for power purposes [76]. Sabido suggested prescribing 3 min rest intervals when performing flywheel squat exercises regardless of the inertial load; conversely, when using 2 min rest intervals, the inertial load should be light [77].

#### 4.2.4. Selected Exercise

This study aligns with the findings of Loren Z., [78] demonstrating that flywheel inertial resistance is particularly suitable for lower limb exercises, such as squats and lunges, because it increases the demand on the hip extensors and ankle plantar flexors while reducing the mechanical demand on the knee extensors. Based on the number of joints involved during exercise, training movements can be classified into single-joint and multi-joint exercises. In our meta-analysis, most researchers selected knee extension as the preferred single-joint exercise, while squats and lunges were the main multi-joint exercises. Anatomically, squats engage multiple muscle groups, including the quadriceps, gluteus maximus, hamstrings, adductors, lower back, core, and calf muscles. Strengthening these muscle groups inevitably enhances lower limb explosive power. Biomechanically, squats require practitioners to control the eccentric lowering phase and quickly overcome resistance during the upward phase, effectively utilizing the elastic potential energy stored during the SSC [79], thereby promoting the growth of lower limb explosive power. Leg extension is a classic method for isolating and training the quadriceps. Studies [40] found that 8 weeks (2–3 days per week) of knee extension using flywheel training technology increased quadriceps femoris muscle hypertrophy by 8%, with similar increases in 1 RM and peak power observed in weight stack resistance exercise. This further confirms the findings of this study.

## 5. Limitations

Recognizing the limitations of this meta-analysis, it is important to note that numerous studies were excluded due to incomplete data or strict inclusion criteria, resulting in a limited number of studies available for subgroup analysis. Most of the investigations included in this review were conducted on male elite athletes, youth athletes, or recreationally trained individuals, thereby limiting the applicability of the conclusions to female and elderly populations. Further research into the effects of FWT on the physical performance of women and older individuals is essential. Another potential shortcoming of this review is the likelihood of selection bias. Our selection was confined to full-text papers published in English, potentially introducing a language bias. Additionally, the diversity in exercise interventions (such as intervention duration, exercises employed, volume, and intensity) possibly significantly influenced the outcomes observed in the meta-analysis. Compounding this, specific details of the training protocols, such as inertial loads, were not consistently documented in the studies reviewed. Despite these constraints, this meta-analysis provides a comprehensive overview of existing research and elucidates, based on scientific literature, the advantages of employing FWT to enhance muscle power.

## 6. Future Research Perspectives

Future studies are necessary to conduct multi-center, large-sample, and long-term randomized controlled trials to provide more reliable evidence-based support for clinical practice. It is recommended that specific characteristics of FWT (volume, intensities, duration, rest interval, and exercise selection) be clearly identified to ascertain the dose–response relationship that maximizes improvements across various demographics, such as different age groups (e.g., adults vs. elders), genders (e.g., male vs. female), types of sports (e.g., volleyball vs. basketball), and athletic levels (e.g., amateur vs. professional). Additionally, exploring the impact of diverse tempo strategies, including accelerated and delayed movement velocity during concentric and eccentric phases, on training outcomes is crucial. Future research also needs to explore load quantification and monitoring in FWT, particularly clarifying the relationship between inertia power and velocity power. Another research direction worth pursuing is the distinct effects of training with different equipment, such as vertical flywheel devices, horizontal flywheel devices, and seated leg curl flywheel devices.

## 7. Conclusions

In conclusion, the results of this systematic review and meta-analysis indicate that isoinertial FWT is an effective tool for enhancing performance aspects closely tied to muscle power, such as countermovement jump and peak power output, in both healthy untrained and well-trained individuals. Moreover, choosing squats and lunges for FWT is more suitable for improving lower limb explosive power. It is recommended that coaches and trainers plan for a six-week period, conducting sessions 2–3 times a week, with an interval of at least 48 h between each session. It is necessary to increase the moment of inertia or shorten rest intervals to enhance training intensity when the total number of sessions exceeds 18. Furthermore, this meta-analysis found that inertial flywheel resistance training is superior to weight stack resistance training in enhancing muscle power, while no significant differences were found when compared to barbell free weights training.

## Figures and Tables

**Table 1:** Basic characteristics of the included studies.

Study (Year)		Characteristics of Participants				Intervention					Results
	N (M/F)	Height (cm)	Weight (kg)	Age (y)	Training Experience	Exercise (Experimental Group Equipment)	Set × Reps	Frequency × Duration = Sessions	Control Group Intensity	Experimental Group Intensity	
[35]	12/12	—	—	69.8 ± 1.3	untrained	knee extension (YoYo flywheel device)	1–4 × 8–12	3 × 12 = 36	80%1 RM	—	MVC ↑ 8%; PT ↑ 28.0% (*)
[36]	15/0	182.8 ± 7.7	91.0 ± 13.8	39.3 ± 8.6	untrained	knee extension (YoYo flywheel device)	4 × 7	2–3 × 5 = 12	7 RM	7 RM	Con PP ↑ 9.0%; ECC PP 12.0%; MVC ↑ 11.6% (*)
[37]	17/0	185.2 ± 8.1	90.0 ± 15.8	39.1 ± 6.6	untrained	knee extension (YoYo flywheel device)	4 × 7	2–3 × 5 = 12	7 RM	7 RM	MVC ↑ 8.1% (*)
[38]	23/0	176.8 ± 3.34	76.8 ± 7.83	22.5 ± 2.5	RT (physically active males)	front step exercise (inertial flywheel device)	5–7 × 8	3 × 6 = 18	8 RM	8 RM	MVC ↑ 11.0%
[39]	29/0	185.0 ± 5.9	83.9 ± 3.9	21.7 ± 2.7	WT (professional handball players)	leg-press (YoYo flywheel device)	4 × 7	2–3 × 6 = 15	7 RM	7 RM	CMJ height ↑ 9.8% (*); PP ↑ 12.9% (*); 1 RM ↑ 12.2%
[40]	8/8	173.0 ± 13.0	79.0 ± 22.0	26.0 ± 4.0	RT (recreationally active individuals)	unilateral knee extension (YoYo flywheel device)	4 × 7	2–3 × 8 = 20	8–12 RM	7 RM	PP ↑ 29.2%; 1 RM ↑ 25.3%
[41]	40/0	180.0 ± 11.0	77.0 ± 5.0	23 ± 4	WT (Italian fourth-division soccer players)	squat (Desmotec flywheel device)	4–6 × 8	1 × 8 = 8	80%1 RM	8 RM	CMJ height ↑ 10%; BS 1 RM ↑ 7%
[42]	32/0	177.6 ± 5.4	75.9 ± 7.6	21.0 ± 1.4	RT (amateur soccer student players)	half squat (Desmotec flywheel device)	3–4 × 5 or 3–6 × 6	2 × 8 = 16	—	5 or 6 RM	CMJ height↑ 4.0%; 1 RM ↑ 9.7%
[43]	8/0	193.5 ± 8.0	87.4 ± 11.7	21.3 ± 3.5	WT (professional basketball players)	half squat (inertial flywheel device)	4–6 × 10	1 × 6 = 6	14 RM	10 RM	CMJ height ↑ (*)
[44]	24/0	190.6 ± 5.9	77.2 ± 7.0	17.6 ± 0.6	RT (junior basketball players)	half squat, Romanian deadlift (isoinertial flywheel device)	2–4 × 8	1–2 × 8 = 12	80%1 RM	8 RM	CMJ height ↑ 11.7% (*); MVC ↑ 18.7%
[45]	16/0	—	93.0 ± 13.1	18.0 ± 1.0	RT (academy rugby union players)	squat, Romanian deadlift, Bulgarian split squat (kbox flywheel device)	4–5 × 6 or 8	2 × 4 = 8	6 RM or 8 RM	6 RM or 8 RM	CMJ PP ↑ 4.0%; CMJ height ↑ 4.9%
[46]	16/0	174.4 ± 7.8	64.5 ± 8.6	15.5 ± 1.2	RT (junior tennis players)	chest press, shoulder press, row, closed stance, and chest crossover (isoinertial flywheel device)	3 × 6 or 8	2 × 8 = 16	50–70% 1 RM	RPE 5–7	CMJ height ↑ 9.7% (*)
[47]	22/0	178.0 ± 1.8	71.5 ± 6.9	21.8 ± 2.7	WT (elite collegiate long-distance runners)	squat (kbox flywheel device)	4 × 7	3 × 6 = 18	85%1 RM	7 RM	CMJ height ↑ 12.0 (*)
[48]	34/0	174.0 ± 7.3	70.5 ± 13.3	16.0 ± 1.4	RT (junior handball players)	lunge, acceleration, squat, single leg hop, and crossover step (isoinertial flywheel device)	3 × 8 or 12	2 × 8 = 16	RPE 6–9	RPE 6–9	UCMJD height ↑ 21.9%
[49]	18/0	184.1 ± 9.7	78.9 ± 10.0	18.6 ± 0.8	WT (elite hockey players)	bilateral/unilateral squat, leg curl, and leg press (isoinertial flywheel device)	3–4 × 6 or 4 × 7	1–2 × 8 = 14	4–12 RM	6 or 7 RM	CMJ height ↑ 5.7%
[50]	11/12	170.0 ± 2.0	73.8 ± 15.9	24.15 ± 3.9	RT (physically active adults)	squat, bench press, deadlift, and row (flywheel training platform)	3 × 4–12	3 × 10 = 30	—	—	MVIT ↑ 11.4

N = number, M = male, F = female, RT = recreationally trained, WT = well-trained, CMJ = countermovement jump, UCMJD = unilateral countermovement jump with dominant leg, PP = peak power, BS = back squat, PT = peak torque, MVC = maximal voluntary contraction, MVIT = Maximal voluntary isometric torque, RM = repetition maximum, RPE = rate of perceived exertion, Con = concentric, and ECC = eccentric, ↑ Statistically significant within-group differences (*p* < 0.05), * statistically significant difference between EOT and CON groups (*p* < 0.05).

## Data Availability

The original contributions presented in the study are included in the article, further inquiries can be directed to the corresponding author.
